# Low Vitamin K Status in Patients with Psoriasis Vulgaris: A Pilot Study

**DOI:** 10.3390/biomedicines12061180

**Published:** 2024-05-26

**Authors:** Simona R. Gheorghe, Tamás Ilyés, Gabriela A. Filip, Ana S. Dănescu, Teodora L. Timiș, Meda Orăsan, Irina Stamate, Alexandra M. Crăciun, Ciprian N. Silaghi

**Affiliations:** 1 Department of Medical Biochemistry, “Iuliu Hațieganu” University of Medicine and Pharmacy, 400349 Cluj-Napoca, Romania; gheorghe.simona@umfcluj.ro (S.R.G.); tamas.ilyes@umfcluj.ro (T.I.); silaghi.ciprian@umfcluj.ro (C.N.S.); 2 Department of Physiology, “Iuliu Hațieganu” University of Medicine and Pharmacy, 400349 Cluj-Napoca, Romania; gabriela.filip@umfcluj.ro (G.A.F.); doratimis@gmail.com (T.L.T.); 3 Department of Dermatology, “Iuliu Hațieganu” University of Medicine and Pharmacy, 400349 Cluj-Napoca, Romania; sorina.danescu@umfcluj.ro; 4 Department of Physiopathology, “Iuliu Hațieganu” University of Medicine and Pharmacy, 400349 Cluj-Napoca, Romania; orasan.meda@umfcluj.ro; 5 Centre Neuchatelois de Psychiatrie, 2000 Neuchâtel, Switzerland; irina.stamate@cnp.ch

**Keywords:** psoriasis vulgaris, vitamin K, vitamin D, osteocalcin, matrix Gla protein, metabolic syndrome

## Abstract

Psoriasis vulgaris (PV) is a disease characterized by skin manifestations and systemic inflammation. There are no published studies to date on vitamin K status assessed by extrahepatic vitamin K-dependent proteins [e.g., osteocalcin (OC) and matrix Gla protein (MGP)] in patients with PV, even if vitamin K was found to promote wound contraction and decrease the healing time of the skin. Metabolic syndrome (MS), a comorbidity of PV, was found to influence vitamin K status, and vitamin D was found to be involved in the pathogenesis of PV. Therefore, our aim was to assess the status of vitamins K and D in subjects with PV. We enrolled 44 patients with PV and 44 age- and sex-matched subjects as a control group (CG), of which individuals with MS were designated the CG with MS subgroup. Furthermore, the PV patients were stratified into two subgroups: those with MS (*n* = 20) and those without MS (*n* = 24). In addition to the quantification of vitamin D and MGP in all subjects, the uncarboxylated OC/carboxylated OC (ucOC/cOC) ratio was also assessed as an inversely proportional marker of vitamin K status. We found an increased ucOC/cOC ratio in the PV group compared to CG but also a greater ucOC/cOC ratio in the PV with MS subgroup than in the CG with MS subgroup. MGP was decreased in the PV with MS subgroup compared to CG with MS subgroup. There was no difference in the vitamin D concentration between the groups. This is the first study to report decreased vitamin K status in patients with PV, independent of the presence of MS.

## 1. Introduction

Representing 90% of psoriasis cases, psoriasis vulgaris (PV) is a chronic inflammatory skin disease that affects approximately 2% of the population [1], with a continually increasing prevalence [2]. It is characterized by the appearance of papulosquamous plaques of various sizes that are well delineated from the surrounding healthy skin. These lesions, from a clinical point of view, are characterized by salmon to pink plaques covered by white or silvery scales. They are predominantly found on the scalp, elbows and knees, and/or lumbosacral region with symmetrical distribution [1]. Considered in the past to be entirely a skin disease, PV is now seen as a systemic condition with a myriad of comorbidities, including cardiovascular disease (CVD), inflammatory bowel disease (IBD), and metabolic syndrome (MS), which can further affect the quality of life and survival of patients [3,4].

The pathogenesis of PV is believed to stem from a synergism between genetics, immunology, and environmental conditions [5]. Linkage analysis identified nine loci (PSORS1-9) for psoriasis susceptibility, of which PSORS1 is the primary determinant and is linked to early onset of the disease [6]. From an immunological point of view, the interplay between innate and adaptive immune cells is responsible for chronic inflammation in the PV [7]. Among environmental risk factors, stress, smoking, alcohol, infections, or certain medications have been implicated in disease development [8].

Along with vitamins D, A, and E, vitamin K belongs to the liposoluble vitamin family. Vitamin K was first described as having a major role in coagulation, receiving its name from the German term “koagulation” [9]. Decades later, after its molecular function was precisely established as a cofactor in the γ-carboxylation reaction of glutamate residues [10], the more accurate origin of the name should likely have been redefined as “karboxylation”.

Several published studies have evaluated the behavior of fat-soluble vitamins, more specifically vitamins A, D, and E, in PV, but none have addressed vitamin K status in this particular disease. Vitamin A is known to have a positive effect on various skin diseases; more specifically, it has a regulatory effect on mast cells, which are known to proliferate with altered functionality in PV [11]. On the other hand, one role of vitamin D at the skin level is the inhibition of keratinocyte proliferation [12], knowing that skin lesions in PV are partially due to keratinocyte hyperproliferation and abnormal differentiation [13]. Vitamin D deficiency has been associated with the pathogenesis of PV [14] and topical treatment with vitamin D analogs has been extensively used in PV [15]. In addition, vitamin E, a potent antioxidant, was found to be deficient in patients with PV compared to healthy subjects [16].

Osteocalcin (OC), the most abundant noncollagen protein found in bones, is a small protein that undergoes posttranslational γ-carboxylation in a process mediated by a γ-carboxylase, which requires vitamin K as a coenzyme. Once γ-carboxylation occurs at its three glutamate residues, they are converted into γ-carboxyglutamic acid, which is accountable for the structure and function of OC in the fully carboxylated state, providing a high affinity for hydroxyapatite, the mineral component of the bone extracellular matrix [17]. Essentially, OC exists in the general circulation as carboxylated OC (cOC) and uncarboxylated OC (ucOC) conformations. Therefore, cOC is detected at high concentrations as a component of the extracellular matrix, while ucOC, which has a lower affinity for calcium, is found in larger quantities in the systemic circulation. Mounting evidence shows that the ucOC/cOC ratio is an inversely proportional proxy used to evaluate vitamin K status [18,19].

The matrix Gla protein (MGP), another extrahepatic protein, is also functionally dependent on vitamin K, more specifically by a γ-glutamate carboxylation reaction, dependent on vitamin K, and needed for its activation. Its major role is to inhibit ectopic calcification, but its involvement in inflammation has also been described [20].

Given that liposoluble vitamins are deficient in PV and play important roles in disease evolution, our objective was to evaluate, for the first time, the vitamin K status in PV in order to improve the understanding of the pathogenesis and treatment options in PV. Moreover, we aimed to evaluate vitamin D status and whether MS influences vitamin K status in patients with PV.

## 2. Materials and Methods

### 2.1. Study Population

This case-control study was conducted between September 2018 and August 2019, enrolling 88 subjects in two groups: a PV group and control group (CG). Within the PV group (*n* = 44), we consecutively included patients with PV selected from the County Emergency Clinical Hospital, a private Medical-Surgical Center and a private dermatology practice in Cluj-Napoca, while the CG (*n* = 44) was comprised of 44 age- and sex-matched volunteers without PV. Subjects with other forms of psoriasis, psoriatic arthritis, neoplasms, other autoimmune or systemic diseases, an immunocompromised system, treatment with prednisone, anti-inflammatory or immunosuppressant medication, vitamin D supplements, or vitamin K antagonists were excluded from the study. After enrollment, all subjects underwent clinical parameter evaluation and laboratory assessment, as described in the present methodology. After the evaluation, 17 subjects from the CG who were declared healthy, without any known medical conditions, were assigned to the healthy control (HC) group. The remaining subjects in the CG (*n* = 27) had either one or a combination of dyslipidemia, hyperglycemia, obesity, or hypertension. Additionally, we stratified the PV group into two subgroups, depending on the presence or absence of MS, as follows: PV with MS (*n* = 20) and PV without MS (*n* = 24). Subsequently, we selected subjects with MS from CG and designated them as the CG with MS subgroup (*n* = 7). The evaluation of MS is presented below in chapter 2.3. Evaluation of Clinical Parameters. All participants signed a written consent form and data processing agreement. Demographic data (age, sex, and place of permanent residence), medical history, comorbidities, and an assessment of daily stress exposure through the Dermatology Life Quality Index (DLQI) [21] were collected as required.

### 2.2. Assessment of Psoriasis Vulgaris

PV was diagnosed following anamnesis, dermatological examination of the skin and visible mucous membranes, and determination of the severity and outspread of disease using the Psoriasis Area Severity Index (PASI) [22]. The diagnosis was performed by experienced dermatologists through clinical examination. To calculate the PASI, the sum of the erythema, infiltration, and desquamation of a single region was multiplied by the numerical value of the region of the body and by the percentage of the region in which the lesion spread. The results obtained for each region are calculated in the PASI. The PASI index values range from 0 to 72 (1.2–57.6). Patients with mild psoriasis had PASI index values up to 10, those with moderate-to-severe psoriasis had PASI values of 10–20, and those with PASI values above 20 were defined as having severe psoriasis.

### 2.3. Evaluation of Clinical Parameters

After enrollment, patients underwent assessment of MS using the International Diabetes Federation (IDF) criteria, in which central obesity is an essential component of MS [23]. According to the IDF definition, patients were categorized as having MS or not having MS. To be classified as having MS, subjects must have central obesity, defined by specific waist circumference (WC) cutoffs (WC cutoffs ≥ 90 cm for men and ≥80 cm for women) plus two or more of the following risk factors: (1) elevated serum triglycerides (≥150 mg/dL) or specific treatment for this lipid abnormality; (2) reduced serum high density lipoprotein (HDL) cholesterol (<40 mg/dL in men/<50 mg/dL in women) or HDL cholesterol disorder treatment; (3) elevated serum fasting glucose (>100 mg/dL) or previously diagnosed type 2 diabetes; and (4) elevated blood pressure [systolic blood pressure (SBP) ≥ 130 mmHg, diastolic blood pressure (DBP) ≥ 85 mmHg] or treatment of previously diagnosed high blood pressure.

A nonstretchable standard tape measure was used to measure WC, determined as the minimum value between the iliac crest and the lateral costal margin, and the value was reported in centimeters (cm).

BP was measured using a mercury sphygmomanometer. Three measurements in the sitting position were performed, involving auscultation of the brachial artery with a stethoscope to detect the appearance and muffling or disappearance of the Korotkoff sounds, which represent SBP and DBP, respectively.

### 2.4. Laboratory Assessments

Fasting blood samples (2 X Vacutainer SSTTM II Advance BD tubes of 7 mL each, containing silica gel) from patients and controls were collected and centrifuged within 2 h of admission, separated into 6 equal volumes, stored in tubes, and immediately frozen at −70 °C until analysis. Blood was separated by centrifugation at 2000× *g* (Rotofix 32A, Hettich Zentrifugen, Mülheim, Germany) for 10 min at 25 °C room temperature.

Serum levels of vitamin D were assessed by enzyme-linked immunosorbent assay (ELISA) using commercially available kits (DIAsource ImmunoAssays S.A., Louvain-La Neuve, Belgium). According to the clinical guidelines, hypovitaminosis D was considered at <20 ng/mL [24]. Human ucOC (Elabscience, Houston, TX, USA) and human cOC (MyBioSource, San Diego, CA, USA) were measured by ELISA, and the ucOC/cOC ratio was calculated as a proxy for vitamin K status. Serum total MGP levels were measured using a sandwich enzyme immunoassay (Elabscience, Houston, TX, USA). Serum levels of high sensitivity C-reactive protein (hsCRP) were measured to aid in the detection and evaluation of inflammation using a chemiluminescence assay (IMMULITE hsCRP, Siemens, Wales, UK). Serum oxidized low-density lipoprotein (oxLDL) levels were measured using a sandwich antibody assay (Elabscience, Houston, TX, USA). Triglycerides and glucose were determined by enzymatic methods using specific reagents for automated equipment (Mindray BS-480, Shenzhen, China).

### 2.5. Statistical Analysis

Statistical analysis was carried out using RStudio Desktop (RStudio © Posit Software, v2023.12.1+402, Boston, MA, USA). A value of *p* ≤ 0.05 was considered to indicate statistical significance at two-tailed levels.

After the analysis of the quantitative data distribution, which was performed by the Shapiro-Wilk test, the majority of the data were found to follow a non-Gaussian distribution, leading to the use of nonparametric tests for uniformity. The data are presented as the median (minimum–maximum). Qualitative data are presented as frequencies. Comparisons of medians were performed by using the Mann-Whitney U test and Kruskal-Wallis test combined with Dunn’s post hoc test. Proportions were analyzed using the chi-squared test. Correlation analysis was carried out using Spearman’s correlation test.

## 3. Results

The current study included 88 subjects, of which 44 were patients with PV and 44 were control subjects without PV, designated as CG. Within the CG, 17 age- and sex-matched individuals who did not have high blood pressure, dyslipidemia, or hyperglycemia were referred to as the healthy control (HC) group. The study population characteristics are presented in Table 1.

**Figure 1:**
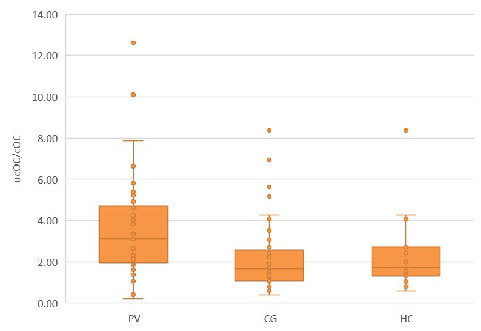
The ucOC/cOC ratio in the PV group compared to CG and HC groups. The median ucOC/cOC ratio in the PV group was greater than that in the CG [3.113 (0.222–12.618) vs. 1.674 (0.371–8.379), *p* < 0.01] and in the HC group [3.113 (0.222–12.618) vs. 1.714 (0.590–8.380), *p* < 0.05].

First, we compared the serum ucOC/cOC ratio among the three study groups. The significant differences in the ucOC/cOC ratios between PV and CG, as well as HC, are depicted in Figure 1.

Furthermore, the significant differences between PV patients with MS and PV patients without MS are summarized in Table 2. The other parameters (vitamin D, oxLDL, hsCRP, MGP, ucOC levels, and the ucOC/cOC ratio) did not significantly differ between the PV subgroups with and without MS; therefore, these parameters are not presented.

**Figure 2:**
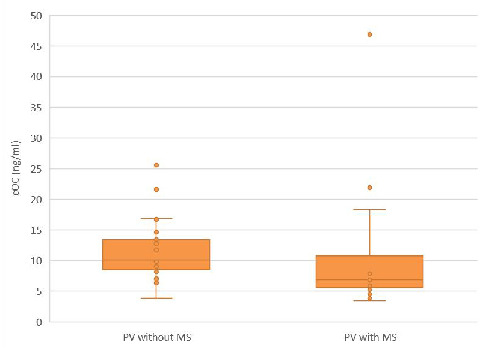
The cOC serum concentrations in the PV subgroups with and without MS. The median cOC in the PV subgroup without MS was higher compared to PV with MS subgroup [10.1 (3.8–25.6) vs. 6.8 (3.4–46.9) ng/mL, *p* < 0.05].

Even if the ucOC/cOC ratio was not significantly different, the cOC levels were significantly higher in the PV with MS subgroup compared to the PV without MS subgroup, as presented in Figure 2.

In the following step, we investigated the differences between PV patients with MS and CG subjects with MS, and the data are shown in Table 3.

**Figure 3:**
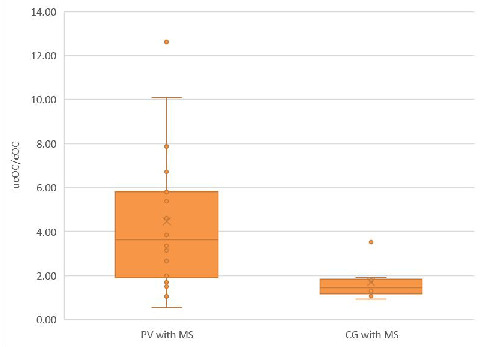
The ucOC/cOC ratio in the PV with MS subgroup was greater than that in the CG with MS subgroup. The median ucOC/cOC ratio in the PV with MS subgroup was greater than that in the CG with MS subgroup [3.63 (0.54–12.62) vs. 1.44 (0.92–3.51), *p* < 0.05].

Moreover, a difference in the ucOC/cOC ratio was also observed between PV patients with MS and CG subjects with MS, as depicted in Figure 3.

Patients who rated their DLQI as having a small impact had significantly lower levels of ucOC (25.6 vs. 35.2, *p* < 0.05) than patients who rated their DLQI as having a moderate impact; however, no other parameter showed any significant differences between patients with small, moderate, or large impacts. No differences or correlations with other parameters were observed when analyzing the PASI score.

Finally, when we analyzed the correlations between parameters, we observed significant correlations only within the PV group, as shown in Table 4.

## 4. Discussion

### 4.1. Vitamin K Status in PV Assessed by ucOC/cOC Ratio

Although other liposoluble vitamins have been studied in PV [25], this is the first study to evaluate vitamin K status in patients with this disease. Given that the ucOC/cOC ratio is a marker of vitamin K status [18] and that its increase is an indicator of vitamin K deficiency [26], our aim was to evaluate the ucOC/cOC ratio in the PV and compare it to that of healthy subjects. We found that the ucOC/cOC ratio was significantly greater and the cOC concentration was lower in subjects in the PV group than in those in the CG group, especially in those included in the HC group. It is known that the carboxylation of OC is dependent on vitamin K, indicating that in these subjects, the availability of the carboxylation cofactor is limited.

For decades, it was believed that PV is a chronic condition affecting only the skin, but recently, it has been postulated that PV is a lifelong systemic inflammatory disorder in which a dysregulated immune system induces overall inflammation [27]. As a consequence of the systemic inflammatory status, a plethora of extracutaneous comorbidities, i.e., CVD, IBD, and MS, have been reported to contribute significantly to the prognosis [3,4].

Vitamin deficiencies are frequent among patients with IBD [28], and in particular, a high prevalence of vitamin K deficiency was reported in patients with Crohn’s disease [29]. The association of PV with IBD can be explained by shared genetic markers and common inflammatory mechanisms [30]. There are two main isoforms of vitamin K: phylloquinone or vitamin K1, which is present in green leafy vegetables and oils, and menaquinones or vitamin K2, which are mainly synthesized by gut bacteria but are also present in fermented foods or dairy products. Because the predominant source of vitamin K is the gut microbiome and dietary vitamin K is absorbed through the intestine, the insufficient vitamin K status in IBD patients is the consequence of bowel inflammation and gut dysbiosis [31]. Interestingly, in addition to the shared inflammation pathways, the gut microbiota pattern in patients with PV is similar to that in patients with IBD. Moreover, vitamin K is produced by Bacteroides [32] appertaining to philum *Bacteroidetes*, a major representative of the human gut microbiota [33], and has been shown to be poorly represented in patients with PV [34]. Considering these previous reports, we can envisage that the vitamin K status is deficient in PV due to the overlapping conditions of intestinal inflammation and an altered microbiome, leading to low levels of cOC and thus an increased ucOC/cOC ratio. However, this hypothesis should be validated through further studies.

Next, with regard to MS, patients were divided into two subgroups (PV with and without MS), and an increase in the ucOC/cOC ratio in the PV with MS subgroup was observed, strengthening our supposition that subjects with PV have vitamin K deficiency. All patients with increased ucOC/cOC ratios had lower cOC concentrations, suggesting a decreased rate of carboxylation, which is vitamin K-dependent. It has been reported that MS lowers vitamin K status, and abdominal obesity is one of the risk factors for MS [35]. As an explanation, it has been proposed that adipose tissue encloses vitamin K, thus reducing its bioavailability and, consequently, lowering the vitamin K status [36]. In our study, we mitigated the influence of MS on vitamin K status by comparing the PV group with MS to the CG group with MS and also finding a significant increase in the ucOC/cOC ratio in the first one. Objectively, it is noteworthy that the sample size of the CG with MS group is rather small, but we consider these findings to be of major interest and they could be further validated by a larger scaled study. Nonetheless, we can state that a poorer vitamin K status is found in PV patients than in healthy controls, and the decrease in vitamin K is independent of the presence of MS. Our findings suggest that the ucOC/cOC ratio could be a robust marker of vitamin K status in PV.

Recently, a new role in the regulation of immunity has been demonstrated for vitamin K, more specifically in reducing the expression of IL-1, IL-6, and TNFα, as well as in decreasing the number and proliferation of T cells [37], which are markers usually involved in the immune vortex found in PV. These additional functions might be pivotal in further explaining the pathogenesis of PV and help in developing new treatments. However, further studies are warranted to validate the role of this immunosuppressant and to establish the underlying physiological mechanism involved.

Moreover, Pazyar et al. [38] demonstrated that topical treatment with vitamin K promotes wound contraction and reduces healing time, a finding that might have beneficial implications for treating PV. Another study showed that the intake of fermented foods containing vitamin K led to a decrease in several inflammatory markers, including IL-6 and IL-12, and to increased microbiome diversity [39]; thus, limiting inflammation would be beneficial for patients with PV. Moreover, several studies have hypothesized that PV treatment should be focused on restoring the gut microbiota, consequently improving vitamin K status [34].

In prospect for future validating studies, we could hypothesize that decreased vitamin K may contribute to the chronic inflammation in PV, an effect that could be limited by dietary vitamin K supplementation and gut microbiota restoration. Moreover, PV patients could benefit from topical treatment with vitamin K due to its role in wound healing.

### 4.2. Matrix Gla Protein in PV: A Vitamin K-Dependent Protein

Subsequently, we analyzed MGP, another vitamin K-dependent protein known to be an important inhibitor of ectopic calcification [40] that is also associated with inflammation [20]. The literature describes five cases of dermal calcification in patients with PV, of which three were on dialysis due to renal failure and two were reportedly without kidney disease. This condition is most frequently associated with chronic kidney disease [41] but also with malignancy, liver disease, vitamin D administration, treatment with vitamin K antagonists and chronic inflammation [42]. It is noteworthy that in these cases, an evaluation of the MGP status would have been compelling. Although scarce, these case reports warrant heightened attention in patient examinations concerning dermal calcification, considering the significant mortality rate of 50% in one year for this complication [43]. Regarding the role of MGP in inflammation, one mouse model study reported that MGP ameliorated intestinal inflammation in colitis [44], while a human study revealed that MGP levels were inversely correlated with the intensity of inflammation in acute pancreatitis [45].

It has long been established that MS is a combination of clinical conditions and is characterized by a proinflammatory state [46,47]. In our study, we detected lower MGP levels in PV patients with MS compared to CG with MS. Moreover, the MGP levels in PV patients were lower than the reference range established in a previous study [48]. By comparing the two groups of subjects in which the constant was PV and the variable was the presence of MS, we observed that the difference in MGP was correlated with the first one. Our finding is in contrast with the only study on MGP in PV, which reported elevated MGP levels in PV and considered MGP to be a protective factor against atherosclerosis, a comorbidity also found in this disease [49]. However, D’Erme et al. [50] reported a downregulated expression of MGP genes in psoriasis lesions compared to healthy skin, a finding consistent with our results. Consequently, PV patients might be at high risk of developing ectopic calcification with subsequent complications; therefore, we can suggest a more thorough assessment in this regard by evaluating calcification scores in PV patients.

### 4.3. Vitamin D in PV

Additionally, we analyzed the vitamin D status in our population and found no differences between the groups. The topic of vitamin D in PV has been widely studied, but the reports are still contradictory. There are studies that found significant vitamin D deficiency in patients with PV, debating whether this deficiency is a risk factor or a consequence of PV [51,52,53], but others have found no differences [54,55]; our results are in accordance with the last category. Moreover, our entire study population was found to have hypovitaminosis D, with the PV group having almost identical median values to those of the healthy subjects. In our opinion, vitamin D deficiency is not related to the studied pathology but is more likely due to the lack of dietary intake and ultraviolet exposure, as well as the high prevalence of vitamin D deficiency demonstrated in the healthy Romanian population [56], factors that could have influenced the vitamin D status between the study groups.

Our research is a pilot study and should be interpreted in this context because one of its limitations is the relatively small sample population, which might not strengthen our results but could be validated by further large-scale studies. Moreover, in future studies, vitamin K status could be determined by assessing the serum concentration of the inactive form of MGP, another marker for vitamin K status. Other shortcomings are the lack of histopathological examination, the ectopic calcification evaluation, and also the disregard of seasonal variations in vitamin D.

## 5. Conclusions

The present pilot study assessed both vitamin K and vitamin D status in patients with PV. Our findings revealed decreased vitamin K status (using the ucOC/OC ratio as a proxy) in patients with PV, while vitamin D levels were not significantly different between patients with PV and those without PV. Moreover, our research indicated that a decrease in vitamin K status is independent of the presence of MS.

Nonetheless, this is the first study to report vitamin K deficiency and decreased MGP levels in PV patients, opening new research opportunities in the pathophysiology and therapeutic management of PV.

## Figures and Tables

**Table 1:** Study population characteristics for quantitative variables.

	PV (*n* = 44)	CG (*n* = 44)	*p*	HC (*n* = 17)	*p*
Age	51 (21–77)	52.5 (20–77)	0.887	51 (24–69)	0.699
WC (cm)	100 (63–150)	87.5 (63–120)	<0.01 *	88 (63–120)	<0.01 *
SBP (mmHg)	130 (110–187)	120 (110–140)	<0.01 *	120 (110–140)	<0.01 *
DBP (mmHg)	80 (65–125)	79 (60–106)	<0.01 *	79 (60–106)	<0.01 *
GLU (mg/dL)	86.5 (41.9–250)	93.3 (41.4–245.2)	0.390	87.9 (70–98.7)	0.897
TRIG (mg/dL)	126.7 (37.7–582.5)	106.3 (35.9–409.3)	0.068	87.9 (46.2–149.1)	<0.01 *
HDL (mg/dL)	45.2 (30.6–106)	49.7 (10.5–81.8)	0.184	64 (44.2–81.8)	<0.01 *
oxLDL (pg/mL)	918 (90.3–2579)	664.2 (6.9–1808)	<0.05 *	743 (6.9–1808)	0.358
hsCRP (mg/L)	2.605 (0.03–100)	2.455 (0.092–58)	0.634	1.27 (0.092–58)	0.171
VitD (ng/mL)	12.4 (2.3–27.9)	13.35 (7–27.3)	0.418	12.1 (7–23.2)	0.923
MGP (pg/mL)	1253 (162.8–1458)	1357.5 (129.7–1503)	0.140	1352 (129.7–1431)	0.618
ucOC (ng/mL)	32.4 (2.2–42.9)	29.9 (3.1–42.9)	0.467	29.9 (11.5–37.2)	0.929
cOC (ng/mL)	8.8 (3.4–46.9)	17.4 (2.9–31.8)	<0.01 *	17.8 (2.9–27.3)	<0.01 *

The data are presented as the median (minimum–maximum). Abbreviations: PV, psoriasis vulgaris; CG, control group; HC, healthy control group; WC, waist circumference; SBP, systolic blood pressure; DBP, diastolic blood pressure; GLU, serum glucose; TRIG, serum triglycerides, HDL, serum high-density lipoprotein; oxLDL, serum oxidized low-density lipoprotein; hsCRP, serum high-sensitivity C-reactive protein; VitD, serum 25-hydroxy-vitamin D; MGP, matrix Gla protein; ucOC, undercarboxylated osteocalcin; cOC, carboxylated osteocalcin; *p* values were calculated versus the PV group. Statistically significant differences are marked with *.

**Table 2:** Variables with significant differences between PV patients with and without MS.

	PV with MS (*n* = 20)	PV without MS (*n* = 24)	*p*
WC (cm)	105 (84–150)	91 (63–125)	<0.01
SBP (mmHg)	135 (110–170)	120 (110–187)	<0.01
GLU (mg/dL)	102.5 (41.9–250)	84.6 (54.2–136.6)	<0.05
TRIG (mg/dL)	157.9 (89.7–582.5)	107.1 (37.7–314.6)	<0.01
HDL (mg/dL)	41.2 (30.6–67)	48.85 (34.8–106)	<0.05

The data are presented as the median (minimum–maximum). Abbreviations: PV, psoriasis vulgaris; MS, metabolic syndrome; WC, waist circumference; SBP, systolic blood pressure; GLU, serum glucose; TRIG, serum triglycerides, HDL, serum high-density lipoprotein. A *p* value <0.05 was considered to indicate statistical significance.

**Table 3:** Significant differences found between the subgroups PV with MS and CG with MS.

Variable	PV with MS (*n* = 20)	CG with MS (*n* = 7)	*p*
WC (cm)	105 (84–150)	80 (78–110)	<0.05
SBP (mmHg)	135 (110–170)	120 (110–138)	<0.05
DBP (mmHg)	102.5 (41.9–250)	76 (65–86)	<0.05
MGP (pg/mL)	157.9 (89.7–582.5)	1468 (875.2–1503)	<0.05
cOC (ng/mL)	41.2 (30.6–67)	20.2 (7–27.9)	<0.05

The data are presented as the median (minimum–maximum). Abbreviations: PV, psoriasis vulgaris; CG, control group; MS, metabolic syndrome; SBP, systolic blood pressure; DBP, diastolic blood pressure; MGP, matrix gla protein; cOC, carboxylated osteocalcin. A *p* value < 0.05 was considered to indicate statistical significance.

**Table 4:** Parameters with significant correlations within the PV group (*n* = 44).

Parameters	Correlation Coefficient	*p*
Age vs. SBP	0.372	<0.05
Age vs. WC	0.3496	<0.05
TRIG vs. HDL	−0.358	<0.05
HDL vs. SBP	−0.315	<0.05
GLU vs. SBP	0.404	<0.01
GLU vs. WC	0.332	<0.05
GLU vs. VitD	−0.404	<0.01
GLU vs. MGP	−0.305	<0.05
SBP vs. DBP	0.592	<0.01
SBP vs. WC	0.555	<0.01
DBP vs. WC	0.354	<0.05
WC vs. hsCRP	0.355	<0.05
ucOC vs. ucOC/cOC	0.414	<0.01
cOC vs. ucOC/cOC	−0.823	<0.01

Abbreviations: WC, waist circumference; SBP, systolic blood pressure; TRIG, serum triglycerides; HDL, serum high-density lipoprotein; GLU, serum glucose; VitD, serum 25-hydroxy-vitamin D; MGP, matrix Gla protein; DBP, diastolic blood pressure; hsCRP, serum high-sensitivity C-reactive protein; ucOC, undercarboxylated osteocalcin; cOC, carboxylated osteocalcin. A *p* value < 0.05 was considered to indicate statistical significance.

## Data Availability

Data are contained within the article.
